# Effects of Backpacks on Ground Reaction Forces in Children of Different Ages When Walking, Running, and Jumping

**DOI:** 10.3390/ijerph16245154

**Published:** 2019-12-17

**Authors:** João P. Barbosa, Mário C. Marques, Henrique P. Neiva, Dulce Esteves, Alicia M Alonso-Martínez, Mikel Izquierdo, Rodrigo Ramirez-Campillo, Cristian Alvarez, Daniel A. Marinho

**Affiliations:** 1 Department of Sport Sciences, University of Beira Interior, 6201-001 Covilhã, Portugal; joaobarbosa02@gmail.com (J.P.B.); henriquepn@gmail.com (H.P.N.); desteves@ubi.pt (D.E.); marinho.d@gmail.com (D.A.M.); 2 Research Center in Sports Sciences, Health Sciences and Human Development, CIDESD, 6201-001 Covilhã, Portugal; 3 Department of Health Sciences, Public University of Navarre, Navarrabiomed, CIBER de Fragilidad y Envejecimiento Saludable (CB16/10/00315), 31006 Pamplona, Navarra, Spain; amalonsoma@gmail.com (A.M.A.-M.); mikel.izquierdo@gmail.com (M.I.); 4 Grupo GICAEDS, Programa de Cultura Física, Deporte y Recreación, Universidad Santo Tomás, Bogotá 110311, Colombia; 5 Laboratory of Human Performance, Quality of Life and Wellness Research Group, Department of Physical Activity Sciences, Universidad de Los Lagos, Osorno 5290000, Chile; r.ramirez@ulagos.cl (R.R.-C.); cristian.alvarez@ulagos.cl (C.A.)

**Keywords:** backpack, children, load-carriage, gait, ground reaction forces

## Abstract

Backpacks for transporting school loads are heavily utilized by children, and their mechanical advantages have been allowing children to transport heavy loads. These heavy loads may increase ground reaction forces (GRFs), which can have a negative effect on joints and bone health. The aim of this study was to investigate the effect of backpacks on the GRFs generated by children during walking, running, and jumping. Twenty-one children from the fifth (G-5, *n* = 9) and ninth (G-9, *n* = 12) grades walked, ran, and jumped over a force plate. When walking, the G-5 had GRF increments in the first (17.3%; *p* < 0.001) and second (15.4%; *p* < 0.001) peak magnitude, and in the total integral of the vertical force (20%; *p* < 0.001), compared to the control condition (i.e., no backpack), and the G-9 had increments of 10.4%, 9%, and 9% (*p* < 0.001), respectively. The G-9 did not prolong their total stance time (*p* > 0.05), unlike the G-5 (*p* = 0.001). When running, total stance time increased 15% (*p* < 0.001) and 8.5% (*p* < 0.001) proportionally to the relative load carried, in the G-5 and G-9, respectively. Peak GRF did not increase in any group when running or landing from a jump over an obstacle. It was found that GRF was affected by the backpack load when walking and running. However, when landing from a jump with the backpack, schoolchildren smoothed the landing by prolonging the reception time and thus avoiding GRF peak magnitudes.

## 1. Introduction

The backpack is the most common strategy adopted by students to carry their school loads [1]. Its use is widespread because it offers several ergonomic and comfort advantages, such as carrying the load near the trunk of the body, which could be symmetrically distributed and leaves the hands free. However, the use of backpacks with heavy loads may induce several modifications in posture and gait, such as reduced pelvic rotation, increments in the head angle, a forward head position, and trunk flexion [2,3,4,5,6,7,8,9,10,11,12,13]. This may lead to adverse effects, such as increments in compression of intervertebral disks and in spine curvatures [1,14,15,16,17,18,19,20].

The use of backpacks may also increase ground reaction forces (GRFs), changing level walking and stair gait patterns [10,21,22,23,24]. In this way, high GRFs have been associated with injuries at the spine level [25] and with lower-limb injuries [23], inducing degradation of the biomechanical properties of the joint cartilage. Mechanical forces influence vertebral growth [26], and high loading rates may have negative effects on bone health [27,28]. In this sense, growing children who use backpacks daily may be at risk, especially if they carry an excessive load, which is the norm [29,30,31].

The scientific community recommends a backpack weight limit of 10% of the child’s body weight (BW) [32,33,34]. However, this limit is often exceeded [30,32]. For example, a recent study found that approximately two-thirds of the Portuguese children analysed, 10 and 15 years of age, carried loads greater than 15% of their body weight at least once a week [29]. Similar results were found by Brzek and colleagues [35] in children from 7 to 9 years old in Poland. This becomes even more disturbing when we realize that younger children carry more weight (normalized to BW) than the older ones. Studies showed that 10-year-old students carried more weight than the 15-year-old students [29], or that students from grades 5 to 8 (ages 8–13) carried more relative weight than those from grades 9 to 12 (ages 13–18) [34]. These loads used by young children, at least until 9 years old, can increase the risk of modifications of posture [35], potentially risking later back pain and/or other health-related issues. Although the scientific literature on this subject is increasing, investigations should be deepened to further understand the effects of improper use of the backpack. In this way, it has already been suggested that research must understand the influence of the loads carried by children of different ages on GRF and all body movement [29].

To our knowledge, previous studies focusing on studying the loads carried by children have been relying on questionnaires to assess pain variables or measuring the effects of hypothetical backpack loads [1,5,8,17,34]. To improve our knowledge on this issue, we thought it important to better understand the effects of using real loads. Therefore, we previously measured the loads carried by the students for a whole week [29] and characterized the backpack weight used by those children that attended the fifth (10 years old) and the ninth grades (15 years old). Backpack load is not the same across the school years [29,35], and children’s physical features also become different as they grow. That is why we considered it important to analyse these two different school grades. The values obtained were normalized to BW and may be used as a reference for the same population, allowing us to further explore other effects, such as the GRFs with a backpack load from a real context.

The few studies that investigated the influence of the carried load on GRFs have focused on walking or stair walking [10,11,22,36]. Nevertheless, children, especially younger children, also play with their backpack on, including running and jumping. Since these are very different kinds of displacements, what would the effect be of backpack transportation on GRFs during these activities? It is commonly known that vertical GRF peaks increase about twice [36] when running or reach 10 times the BW [37] when landing from a jump. Therefore, will backpack transportation increase this even more? Knowing that high GRF levels could potentially develop adverse health issues [13,22,27], it is important to understand whether they are increased by the loads carried by children in their usual activities. Therefore, the aim of this study is to analyse the effect of backpack transportation on GRFs in children during walking, running, and jumping, in two different school years.

## 2. Materials and Methods

### 2.1. Participants

Twenty-one children participated in this study, considering the possibility of the school timetable and personal availability. The participants attended the ninth grade (G-9; five females with mean age of 14.82 ± 0.23 years, 55.20 ± 7.09 kg of body mass, 1.57 ± 0.06 m of height, and 22.54 ± 3.20 kg/m^2^ of body mass index; and seven males with mean age of 15.16 ± 0.97 years, 57.71 ± 14.69 kg of body mass, 1.67 ± 0.09 m of height, and 20.52 ± 3.92 kg/m^2^ of body mass index) and the fifth grade (G-5; four females with mean age of 11.08 ± 0.40 years, 34.25 ± 5.38 kg of body mass, 1.40 ± 0.08 m of height, and 17.70 ± 4.65 kg/m^2^ of body mass index; and five males, with a mean age of 10.88 ± 0.33 years, 36.20 ± 9.18 kg of body mass, 1.42 ± 0.06 m of height, and 17.68 ± 3.27 kg/m^2^ of body mass index). According to the classification of the education system of the country, the fifth grade represents the first year of the second cycle of the basic education system and the ninth grade represents the last year of the third cycle and the last year of basic education. Students in a non-regular school programme and others in a situation that could affect the backpack content were excluded from the analysis. All children and parents/guardians were informed about the experimental procedures of the study, and after acceptance, the parents/guardians signed the informed consent. This study was conducted in accordance with the International Charter for Ethical Research Involving Children. The data collection was approved by the school principal and the Research Centre in Sports Sciences, Health Sciences, and Human Development at the University of Beira Interior Review Board approved study procedures, in accordance with the Declaration of Helsinki.

### 2.2. Data Collection

The experimental sessions took place in the sports equipment storage room of the school sports gym. This room was spacious enough to install all the apparatus, had natural light, and offered privacy, avoiding external influences. Each participant performed the assessments in a single session, so all the tasks could be performed in the same conditions (temperature: 20.8 ± 0.6 °C humidity: 54.0% ± 2.0%).

Participants were asked to perform all the evaluations on a wood platform, with 330 cm length, 62 cm width, and 6 cm height. A force plate (MuscleLab, Ergotest Innovation; Porsgrunn, Norway), with 80 cm length, was embedded in the middle of the wood platform. The software Ergotest MuscleLab V8.0 (Ergotest Innovation A.S., Porsgrunn, Norway) was linked to the force platform in order to collect and export data. Considering that the purpose was to analyse the effect of the backpack on GRF, both conditions (loaded and unloaded) were performed so they could be compared. All the participants were requested to perform the tests with their usual clothes and shoes, that is, what they wear when carrying their backpack to school. They presented themselves with varied clothes, but all used sports shoes.

At the beginning of each session, participants were asked to walk, run, and jump over the platform to familiarize themselves with the equipment, until they demonstrated similar performance on the platform as they did on the ground. All participants stepped on the force plate with the right foot (dominant) during their walking and running performance. Therefore, they were asked to set their individual starting point so they could walk and run normally and with no need to adjust the movement to step on the GRF platform with the right foot. Subsequently, to familiarize themselves with the jumping task, they jumped with both feet over a cardboard box (23 cm height, 38 cm length, and 51 cm width), landing on the GRF platform several times. All these preparation tasks were done with and without a backpack. Then, the participants rested for 45 min, so that any fatigue effect could be avoided.

The loads carried on the backpack were the mean loads usually carried by these children for each of the school years, as previously reported [29]. The backpack was carried with 5 kg of books by G-5 children and with 4.5 kg by the G-9 children. It was placed over both shoulders and individually adjusted. Adjustment backpack straps were loosened, and the participants were asked to adjust them as they usually do with their backpacks. The backpack used in this study was the Padded model, from the brand Eastpack (Boston, MA, USA), one of the most used models by children in the region where the study was conducted.

To avoid learning or fatigue effect, the order of task performance was totally randomized using the randbetween function on Microsoft Excel. Each task was evaluated for five repetitions. Each session was conducted with three participants, and the resting time between tasks was the necessary time for the other two participants to complete their tasks. As the performance order was totally randomized, the first participant could perform his five repetitions of “walk with backpack”; the second “run unloaded”; and the third “jump loaded”, for example. Then, a new cycle started, until all tasks were performed by all participants. No performance feedback was provided to the participants during the recording trials.

### 2.3. Data Analysis

**Figure 1:**
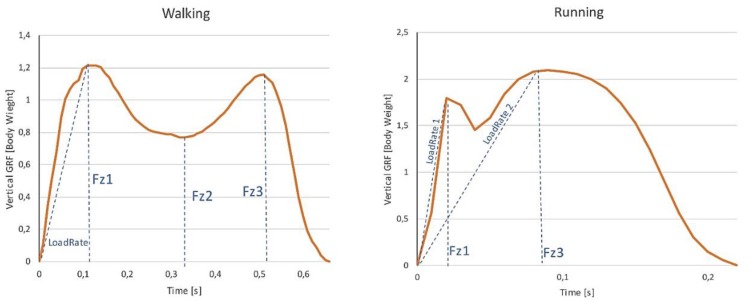
Typical vertical ground force (GRF)/time curves from one of the participants during unloaded walking and running, demonstrating the first peak (impact force peak—Fz1), relative minimum (Fz2), the second peak (Fz3), and the loading rate (LoadRate), normalized to body weight.

Microsoft Excel (Microsoft Office 365 ProPlus) was used to randomize conditions of performance order with the randbetween function and to organize data exported from MuscleLab. The variables analysed were related to the time of support (total or between phases of support) and vertical forces: absolute maximum (MaxAbsl); peak values of each phase of support (Fz1, Fz2, Fz3); integral of forces (or impulse); and loading rate (LoadRate). Magnitudes of force variables were also calculated in the function of the BW of the subjects. The relative effect of the backpack (BPackW) was calculated by dividing the difference between loaded and unloaded by the backpack weight. Figure 1 can be observed as a typical force/time curve obtained during data collection in walking and running situations.

To analyse data results, descriptive statistics, the T-Test for paired samples, the T-Test for independent samples, normality tests, and intraclass correlation coefficient were performed. As the main goal was to compare the tasks performed with and without a backpack on each subject, the T-Test for paired samples was the main statistical operation. The T-Test for independent samples was used only to compare the relative effect of the backpack between G-5 and G-9 on the walking GRF peaks. Statistical procedures were performed using the Statistical Package for the Social Sciences (SPSS v.20) (IBM, Corp., New York, USA), and the statistical significance was set at *p* < 0.05. Additionally, the effect size of the differences verified on T-Tests was calculated based on Cohen’s d method, using the formula for paired samples proposed by the GPower project [38]. As originally proposed by Cohen [39], the interpretation of the effect sizes was considered small when 0.2 ≤ d < 0.5; medium when 0.5 ≤ d < 0.8; and large when d > 0.8.

## 3. Results

Good-to-excellent inter-trial repeatability was found for all variables (ICC > 0.90) on the walking and running situations. When walking, the loads that students usually carry to school increased stance time and deeply affected all force variables analysed. In the G-5, the first peak magnitude increased 17.3%, the second peak magnitude increased 15.4%, and the relative minimum increased 14% with the backpack on. The total integral of vertical force increased by 20%. The rate of increasing the vertical force from the first touch on the ground until the first peak increased by 11.1%. In this group, the total stance time increased mainly by time between peaks, and probably with higher importance of the first half, which is the time between the first peak until reaching the minimum value of vertical force (Table 1).

Older students (G-9) carried less weight, specifically about 8% of the BW. There were no significant differences in the time variables between the use or non-use of a backpack. However, as with younger students, the presence of the load on the backpack increased all force variables. The first peak increased by 10.4%, and the second peak was incremented at 9%. On the contrary, the increment on the relative minimum was only 3%, which represents only 28% of the added load. The total integral of forces increased by 9%. The loading rate until the first peak was incremented on 360 N/s (7.2%) (Table 1).

When running with a backpack on, stance time increased 15% in fifth-grade students and 8.5% in ninth-grade students. Force variables were affected differently than in walking: peak values were not increased, and the rate of force applied decreased. Only the integral of force increased—14% and 9%—yet less than when walking (Table 2).

When landing from jumping over the obstacle with both feet at the same time, the integral of force increased for both; however, remaining variables were affected distinctly in fifth-grade students and in ninth-grade students: no differences were observed in fifth-grade students, while in ninth-grade students, the landing time increased, and force peak decreased (Table 3).

## 4. Discussion

The current study aimed to investigate the influence of the load carried in school backpacks on GRFs. It was our intention to analyse real daily conditions—that is, to analyse the impact of loads that children carry to school in a real context. The loads carried had been previously assessed, and thus we could use the loads that are normally carried by children who were attending fifth grade and ninth grade at a public school [29]. It was found that generally, carrying these loads affected GRFs when walking and running. When walking, the peak values of each phase of support, integral of forces, and loading rate were increased with a backpack on, in both groups. However, these force variables were affected differently in running. In this situation, the peak values were not increased, and the rate of force decreased for both groups. When landing from jumping, the integral of force was increased for both groups, but the landing time increased and force peak decreased only in ninth grade students.

The findings were consistent with several other studies that compared unloaded with loaded walking [6,10,21,22,23,40,41,42]. To our knowledge, no studies have done this analysis on another means of locomotion beyond walking. The studies reported walking speed decreases [6,10,37,38], increased magnitudes of vertical GRFs [21,22,23,36,37], and a protective behaviour by the loaded participants [10,21] in order to attenuate the increments of the vertical GRF magnitudes. In this investigation, we found these previously reported effects, but they were expressed differently based on the student’s school level and types of locomotion.

When walking, the use of a backpack by fifth-grade students induced a prolongation of the stance time, which is in line with the data recorded by Ahmad and colleagues [42], when 7–9-year-old children carried a backpack. We also registered a significant increment of the force peaks and the integral of forces when the backpack was carried. The first peak magnitude increased by 17%, and the second peak increased 15% in the G-5. Mosaad and colleagues [22] registered an increment of 11% of the first peak by 10-year-old children when carrying 7.5% of their BW. It should be noted that the children in the current study carried about 14.3% of their BW. Razali and colleagues [41] registered increments of 10.4% and 25% when children of 9 to 10 years of age carried the backpacks with 10% and 15% of their BW, respectively. Despite the high increase in the first peak force magnitude, the loading rate increased only 11%, which means a low increment. This could be because of the prolongation of the stance time that attenuated the loading rate increase. As observed by Simpson and colleagues [21], with increased load, children unconsciously adjust gait features, namely, the speed and the knee flexion. This attenuation could be framed as a protective behaviour, adapting the gait pattern in consequence of load carriage, and aiming to minimize possible harmful effects of high vertical GRFs over the musculoskeletal system, as suggested by Castro and colleagues [10]. The total integral influenced both by the increment in force peaks and the prolongation of the stance increased 20% with the backpack on in the G-5 when walking.

The ninth-grade students did not prolong their stance time in the walking situation. However, they also had large increments in the first and second force peaks. The 10% increment of the first peak could be compared to the 11% increment verified by Mosaad and colleagues [22], as reported above, with the children carrying a similar relative load. While unloaded, the magnitude of the first peak was about 1.25 BW for every student, which is in an expected range of values [36]. However, when a backpack is added, the magnitude of the first peak increased by a factor of 1.5 (G-5), or 1.6 times (G-9) the load added. Therefore, the load added in the backpack had double the influence than if that load was added as BW. That means that the magnitude of the first force peak of a 50 kg student with a backpack of 5 kg should be higher than that of a 55 kg student. This is probably due to the modifications caused by posture and gait patterns [22], as the backpack is not fully attached to the body. Accordingly, the first force peak of G-5 could be calculated through the equation 1.25 × BW + 1.53 × BackpackWeight and, in the case of G-9, the equation should be 1.26 × BW + 1.65 × BackpackWeight. Applying this analysis to the data provided by Mosaad and colleagues [22], where 10-year-old children carrying 7.5% BW were studied, we obtain the formula 1.12 × BW + 1.60 × BackpackWeight. Therefore, similar levels of the relative effect of the backpack were found on the first peak of GRF.

Curiously, as it is possible to ascertain above, the influence of the backpack on the first peak was higher on G-9 (measured as the percentage of change). As G-5 carried a greater relative load, they had the need to slow down the displacement, more so than the G-9, which is in agreement with previous studies where greater relative loads induced greater decreases in walking speed and increases in support time [6,42]. For example, Ahmad and colleagues [42] found significant and moderate main effects of the load on the velocity and duration of the stance, for loads of 10% BW and 15% BW on children of 7–9 years old. Therefore, as higher speeds lead to higher GRF peak value increments [36,43], this could explain the greater relative influence of the backpack among G-9. Following this analysis, in the second peak, the factor of increment was about 1.3 times the load added, for both groups. In this case, the walking speed had no influence, as expected. According to Schwartz and colleagues [43], walking speed affects the first peak more than it affects the second peak.

Comparing again to the data provided by Mosaad and colleagues [22], the increment on the second peak was also 1.3 times the load added. So, the relative influence of the backpack load was very similar to that found in this study, especially on G-9. Remember that the children carried 7.5% of their BW, close to our G-9 (8% of BW). This may indicate the possibility of predicting the effect on GRF peaks by knowing the backpack load, at least on the situations framed in the “comfort zone”, where children do not need to adjust gait features.

If, when walking, all force variables increased with added load, when running, the findings were different. Peak forces did not increase, and the loading rate decreased. This is probably due to the significant decrease verified in speed, evidenced by the increment in stance time. When running, G-5 increased the stance time in about 15% of unloaded condition, while G-9 increased by about 8% the unloaded time. Interestingly, there were similar values of the relative load carried, which may suggest that children had the need to slow down the movement in the same proportion of the load carried. Silder and colleagues [44] verified a smaller increase in the standing time, but also verified an increase in vertical GRF peak in adult runners wearing a weight vest. In the present study, children prolonged their stance in order to totally avoid the GRF increment, evidencing the protective effect described by Castro and colleagues [10] for walking or simply to contain the backpack’s oscillation. When running, because it is not fully attached, the backpack tends to maintain a desynchronized movement with the body.

When the children were jumping over a paper box with both feet at the same time, the backpack induced obviously visible modifications to the movement. The increment on the knee flexion was obvious and originated a 20% prolonged reception, so they can keep GRF magnitudes acceptable.

Some limitations of the current study should be addressed. We acknowledge that this study was performed for students who follow a specific national curriculum at a public school, and the results might be affected by the specific conditions of the school, city, and country. The current data should be interpreted within the context of the study and its sample of young students. The primary limitation of this research is the limited number of subjects. There are inherent difficulties in randomizing some individuals when attempting to investigate young students at a public school. Because of the small sample size, we tried to understand the effects of using a backpack in males and females together. In this way, we increased the statistical power of the data results, and consequently, it was possible to obtain more reliable conclusions. We should not disregard the possible existence of different maturational status of females or/and males, mainly in G-9. This is usually a confounding factor in research, but in this specific case, it was not considered relevant. According to the main purpose of the study, we performed a within-subject analysis. This meant that each subject was compared with himself or herself, regardless of maturational status, and/or sex, removing any possible influence of these factors. Future studies should increase the number of participants and analyse different ages and maturational statuses. This would allow us to deepen the analysis of the data. Moreover, studies should also look further into the physiological response of carrying a backpack and should try to find strategies (i.e., by changing the design of the backpack) to reduce the impact of carrying it.

## 5. Conclusions

The loads carried by the Portuguese students in their backpacks significantly affected GRFs. When walking, the load carried induced significant increments in GRF peaks and integral of force, with superior effect sizes on the fifth-grade children. In both groups, the increment in GRF peak magnitude was more than 1.5 times the load added (or 150% of the load added). When running, load-bearing students increased stance time on the proportion of the relative load carried, avoiding GRF peak magnitude increases. School children also succeeded in avoiding GRF peak magnitudes when landing from a jump with the backpack, smoothing the landing by prolonging the reception time by about 20%.

## Figures and Tables

**Table 1:** Time and force variables (mean ± standard deviations) for loaded and unloaded walking.

Walking	G-5				G-9			
	Unloaded	Loaded	*p*	*d*	Unloaded	Loaded	*p*	*d*
**Time variables**								
Total stance time (s)	0.61 ± 0.06	0.63 ± 0.05	0.001	0.55	0.65 ± 0.04	0.66 ± 0.04	0.077	
Time to Fz1(s)	0.13 ± 0.02	0.14 ± 0.02	0.188		0.14 ± 0.05	0.14 ± 0.01	1.000	
Time from Fz1 to Fz2 (s)	0.16 ± 0.03	0.17 ± 0.02	0.088		0.16 ± 0.07	0.17 ± 0.02	0.391	
Time from Fz2 to Fz3 (s)	0.18 ± 0.03	0.178 ± 0.05	0.696		0.18 ± 0.03	0.18 ± 0.02	0.925	
Time after Fz3 (s)	0.15 ± 0.02	0.15 ± 0.02	0.161		0.17 ± 0.03	0.17 ± 0.02	0.855	
Time between peaks (s)	0.33 ± 0.04	0.34 ± 0.04	0.009	0.40	0.34 ± 0.08	0.35 ± 0.02	0.437	
**Force variables**								
First peak-Fz1 (N)	433.01 ± 108.29	507.98 ± 130.27	<0.001	1.50	695.74 ± 128.03	768.37 ± 150.18	<0.001	1.14
First peak-Fz1 (N.BW^−1^)	1.24 ± 0.11	1.46 ± 0.14	<0.001	1.62	1.26 ± 0.14	1.39 ± 0.16	<0.001	1.19
Fz1 BPackW (N.BW^−1^)		1.53 ± 1.26				1.65 ± 1.45	0.030 ^#^	
Fz2 (N)	217.99 ± 51.87	248.56 ± 44.67	<0.001	0.78	379.96 ± 86.17	392.07 ± 89.62	0.049	0.26
Fz2 (N.BW^−1^)	0.63 ± 0.10	0.73 ± 0.13	<0.001	0.89	0.68 ± 0.08	0.71 ± 0.09	0.022	0.30
Second peak-Fz3 (N)	417.39 ± 63.57	481.65 ± 94.61	<0.001	1.25	650.18 ± 116.30	708.46 ± 131.45	<0.001	1.27
Second peak-Fz3 (N.BW^−1^)	1.22 ± 0.10	1.40 ± 0.10	<0.001	1.52	1.18 ± 0.07	1.28 ± 0.09	<0.001	1.34
Fz3 BPackW (N.BW^−1^)		1.31 ± 0.16				1.32 ± 0.13		
MaxAbsl (N)	446.06 ± 98.22	520.92 ± 125.04	<0.001	1.79	709.81 ± 122.55	774.42 ± 145.19	<0.001	1.07
MaxRelative (N.BW^−1^)	1.29 ± 0.10	1.50 ± 0.11	<0.001	2.33	1.3 ± 0.16	1.42 ± 0.19	<0.001	1.11
Total integral (N.s)	167.79 ± 37.35	201.06 ± 43.95	<0.001	2.64	289.4 ± 62.75	314.86 ± 66.83	<0.001	1.37
LoadRate (kN.s^−1^)	3.42 ± 1.05	3.80 ± 1.12	0.023	0.35	5.00 ± 0.98	5.36 ± 1.05	0.017	0.32

BPackW = (loaded − unloaded)/backpack weight; (N/BW) = normalized to bodyweight. Fz1 = first vertical force peak; Fz2 = vertical force relative minimum; Fz3 = second vertical force peak; MaxAbsl = vertical force absolute maximum; ^#^ *p* level for the comparison between G-5 and G-9.

**Table 2:** Time and force variables (mean ± standard deviations) for loaded and unloaded running.

Running	G-5				G-9			
	Unloaded	Loaded	*p*	*d*	Unloaded	Loaded	*p*	*d*
**Time variables**								
Total stance time (s)	0.26 ± 0.03	0.30 ± 0.03	<0.001	1.61	0.30 ± 0.03	0.32 ± 0.03	<0.001	0.94
Time to Fz1 (s)	0.03 ± 0.01	0.04 ± 0.01	0.017	0.37	0.04 ± 0.01	0.04 ± 0.01	0.056	
Time to Fz2 (s)	0.11 ± 0.02	0.13 ± 0.02	<0.001	0.74	0.12 ± 0.02	0.14 ± 0.02	<0.001	0.76
Time between peaks (s)	0.08 ± 0.01	0.09 ± 0.02	0.001	0.55	0.09 ± 0.01	0.10 ± 0.02	<0.001	0.66
**Force variables**								
First peak-Fz1 (N)	502.77 ± 191.88	486.97 ± 152.16	0.416		729.75 ± 210.28	729.3 ± 219.93	0.984	
First peak-Fz1 (N.BW^−1^)	1.46 ± 0.43	1.41 ± 0.34	0.444		1.36 ± 0.32	1.35 ± 0.28	0.811	
Second peak-Fz2 (N)	854.61 ± 159.96	835.51 ± 170.55	0.105		1395.71 ± 263.7	1405.72 ± 290.71	0.396	
Second peak-Fz2 (N.BW^−1^)	2.49 ± 0.19	2.42 ± 0.18	0.051		2.63 ± 0.52	2.64 ± 0.48	0.723	
MaxAbsl (N)	854.61 ± 159.96	835.9 ± 170.03	0.110		1395.71 ± 263.7	1405.72 ± 290.71	0.396	
Max Relative (N.BW^−1^)	2.44 ± 0.19	2.37 ± 0.17	0.054		2.58 ± 0.51	2.59 ± 0.47	0.726	
Load Rate 1 (kN.s^−1^)	16.85 ± 10.25	14.51 ± 7.41	0.058		23.35 ± 10.85	20.78 ± 7.28	0.019	0.31
Load Rate 2 (kN.s^−1^)	7.71 ± 1.34	6.76 ± 1.68	<0.001	0.56	11.51 ± 2.76	10.59 ± 2.84	<0.001	0.53
Total integral (N.s)	113.83 ± 31.05	129.99 ± 30.34	<0.001	1.68	203.32 ± 41.29	222.22 ± 44.77	<0.001	1.26

BPackW = (loaded − unloaded)/Backpack Weight; N.BW^−1^ = normalized to bodyweight. Fz1 = first vertical force peak; Fz2 = vertical force relative minimum; Fz3 = second vertical force peak; MaxAbsl = vertical force absolute maximum.

**Table 3:** Time and force variables (mean ± standard deviations) for loaded and non-loaded jumping.

Jumping	G-5				G-9			
	Unloaded	Loaded	*p*	*d*	Unloaded	Loaded	*p*	*d*
**Time variables**								
Total reception time (s)	0.24 ± 0.16	0.29 ± 0.19	0.102		0.34 ± 0.17	0.42 ± 0.139	0.000	0.49
Time to max (s)	0.04 ± 0.02	0.04 ± 0.02	0.847		0.06 ± 0.02	0.061 ± 0.02	0.050	0.26
**Force variables**								
MaxAbsl (N)	1783.90 ± 580.90	1761.40 ± 627.50	0.786		2474.50 ± 804.30	2269.20 ± 659.40	0.011	0.34
Max Relative (N.BW^−1^)	5.14 ± 1.37	5.05 ± 1.43	0.699		4.49 ± 1.24	4.12 ± 1.00	0.011	0.34
LoadRate (kN.s^−1^)	61.25 ± 42.34	61.68 ± 39.52	0.944		58.26 ± 49.05	46.69 ± 40.57	0.072	
Total integral (N.s)	144.25 ± 92.60	178.52 ± 104.08	0.025	0.34	308.97 ± 149.92	387.96 ± 138.61	0.000	0.62

N.BW^−1^ = normalized to bodyweight. MaxAbsl = vertical force absolute maximum.
